# Peri-implantation lethality in mice carrying megabase-scale deletion on 5qc3.3 is caused by Exoc1 null mutation

**DOI:** 10.1038/srep13632

**Published:** 2015-09-08

**Authors:** Seiya Mizuno, Kohei Takami, Yoko Daitoku, Yoko Tanimoto, Tra Thi Huong Dinh, Saori Mizuno-Iijima, Yoshikazu Hasegawa, Satoru Takahashi, Fumihiro Sugiyama, Ken-ichi Yagami

**Affiliations:** 1 Laborarory Animal Resource Center, University of Tsukuba, 1-1-1 Tennodai, Tsukuba, Ibaraki 305-8575, Japan

## Abstract

We found a novel spontaneous mouse mutant with depigmentation in the ventral body, which we called White Spotting (WS) mouse. Genetic investigation revealed deletion of a > 1.2-Mb genomic region containing nine genes (*Kit*, *Kdr*, *Srd5a3*, *Tmeme165*, *Clock*, *Pdcl2*, *Nmu*, *Exoc1*, and *Cep135*). We designated this mutant allele *Kit*^*WS*^. Interestingly, homozygous mutants (*Kit*^*WS/WS*^) showed a peri-implantation lethal phenotype. Expression analyses of these nine genes in blastocysts suggested that *Exoc1* was a prime candidate for this phenotype. We produced *Exoc1* knockout mice, and the same peri-implantation lethal phenotype was seen in *Exoc1*^−/−^ embryos. In addition, the polygenic effect without *Exoc1* was investigated in genome-edited *Kit*^*WE*^ mice carrying the Mb-scale deletion induced by the CRISPR/Cas9 system. As *Kit*^*WE/WE*^ embryos did not exhibit the abnormal phenotype, which was seen in *Kit*^*WS/WS*^. We concluded that peri-implantation lethality in *Kit*^*WS/WS*^ was caused by a monogenic defect of *Exoc1*.

There have been many reports of spontaneous mouse mutants with depigmentation phenotypes^1^, among which *Kit* gene mutant mouse strains have been investigated in detail. More than 150 *Kit* mutant strains, including 72 spontaneous mutants, are listed in the Mouse Genome Informatics (MGI) database (http://www.informatics.jax.org). The Kit protein consists of an intracellular tyrosine kinase domain and an extracellular domain containing five immunoglobulin-like repeats^2^. The *Kit*^*W*^ gene, with a spontaneous point mutation resulting in the skipping of a critical exon, encodes a non-functional Kit protein lacking the extracellular domain^3^. Mice heterozygous for the *Kit*^*W*^ mutation (*Kit*^*+/W*^) show depigmentation in the ventral body, and *Kit*^*W/W*^ homozygotes die around the first week of life^4^. Several large-scale mutations have been found around the *Kit* gene region. The *Kit*^*W–57J*^ allele shows a deletion of approximately 80 kb in the upstream region of the *Kit* gene^5^. Moreover, megabase (Mb)-scale mutations were found in *Kit*^*W–bd*^ (2.8 Mb inversion)^5^ and *Kit*^*W–19H*^ (>3.5 Mb deletion)^6^. More than 20 genes, including *Kit*, are completely deleted in the *Kit*^*W–19H*^ allele. Interestingly, *Kit*^*W–19H/W–19H*^ mice die at the peri-implantation stage. Therefore, one or more of these 20 genes is/are thought to be the gene(s) responsible for this phenotype. However, the causative gene has not yet been conclusively identified.

The exocyst complex plays a critical role in exocytosis, which is a vesicle transportation event involved in provision of plasma membrane proteins and lipids to the plasma membrane and secretion of soluble vesicle components, such as hormones and cytokines. The exocyst complex is composed of eight proteins: Exoc 1, 2, 3, 4, 5, 6, 7, and 8^7^. As several exocyst complex-related proteins, such as Arp3 and desmosomal proteins (Dsp, Dsc3, and Dsg2), are essential for early embryo development^8^,^9^,^10^,^11^, exocyst components are predicted to play important roles at the early embryonic stage. The *in vivo* functions of only two of eight exocyst components have been investigated in gene-modified mouse models. *Exoc4* null mutant mice showed embryonic lethality at embryonic day 10.5 due to abnormal mesoderm formation^12^. In addition, *Exoc6* spontaneous mutant mice exhibit severe microcytic anemia^13^. However, the *in vivo* functions of the other exocyst components are unclear.

Here, we report a *de novo* spontaneous Mb-scale mutant allele, *Kit*^*WS*^, with a genomic deletion of at least 1.2 Mb containing nine genes (*Kit*, *Kdr*, *Srd5a3*, *Tmeme165*, *Clock*, *Pdcl2*, *Nmu*, *Exoc1*, and *Cep135*). As *Kit*^*WS/WS*^ showed a peri-implantation lethal phenotype, we attempted to identify the causative gene for this phenotype. Gene expression analysis in blastocysts suggested *Exoc1* as the first candidate gene. We produced and analyzed an *Exoc1* knockout mouse strain to determine the *in vivo* function of this gene. As expected, the abnormal phenotype of *Exoc1* null embryos was the same as that of *Kit*^*WS/WS*^. Finally, we introduced the Mb-scale deletion allele (the region from *Kit* to *Nmu*) in mice using the CRISPR/Cas9 system. This introduced mutation did not affect early embryonic development. These data indicate that *Exoc1*, which is located in the vicinity of the *Kit* gene, is the monogenic causative gene for peri-implantation lethality.

## Results

### Appearance of the novel natural mutant with depigmentation

**Figure 1:**
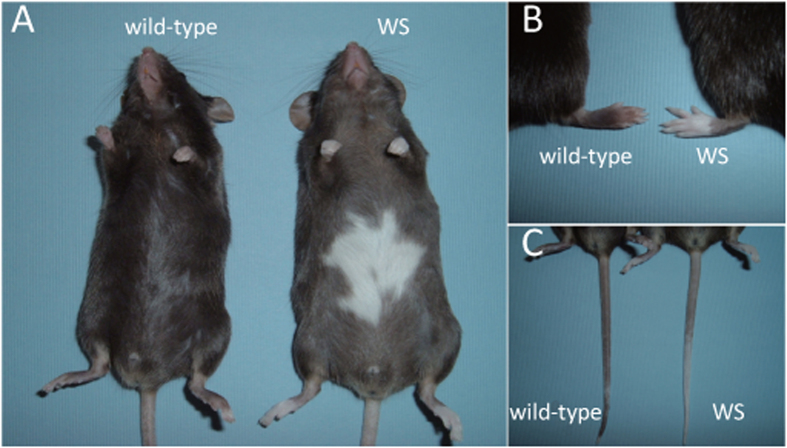
Depigmentation in WS mouse. Depigmentation phenotypes in the ventral body (**A**) and the tips of the feet (**B**) and tail (**C**) were seen in WS mice.

A male mouse generated from a pair of C57BL/6J (B6J) obtained from Charles River Laboratories Japan exhibited depigmentation in the ventral body (Fig. 1A) and the tip of the tail and foot (Fig. 1B,C). We called this White Spotting (WS) mouse. These abnormalities were not observed in its male or female siblings. To examine whether the abnormal pigmentation was heritable, we carried out test crosses between the WS male and wild-type females. Of 20 progeny, nine mice (3 females and 6 males) showed depigmentation, which suggested that the abnormal phenotype is inherited in an autosomal dominant manner.

### Identification of deleted genome region

**Figure 2:**
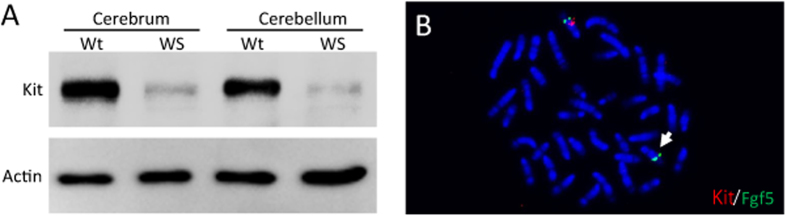
*Kit* gene expression and location. (**A**) Kit protein was detected on Western blotting of the cerebrum and cerebellum. The signals of Kit in WS mice were weaker than in wild-type. (**B**) FISH to metaphase chromosome spreads of WS mice. FITC (green) signals, which reflected the *Fgf5* gene locus, were observed on both copies of chromosome 5. In contrast, Cy3 (red) signals, reflecting the *Kit* gene locus, were only seen on one copy (white arrow).

As *Kit* gene mutant mouse strains were reported to show depigmentation with an autosomal dominant inheritance pattern^14^,^15^, we postulated that the abnormal pigmentation in WS mice is caused by a *de novo Kit* gene mutation. Kit protein expression in WS was confirmed by western blotting. As Kit is expressed in the brain^16^, we examined Kit expression in the cerebrum and cerebellum from wild-type and WS adults. As expected, very weak Kit signals were observed in WS mice (Fig. 2A). Next, we performed fluorescence *in situ* hybridization (FISH) to investigate genomic mutation on the *Kit* gene locus (5qc3.3). Bacterial artificial chromosome (BAC) DNA vectors were used as probes. The BAC RP23-142L11 (derived from Chr. 5: 75,533,600–75,737,738), which carries the full-length *Kit* gene region (Chr. 5: 75,574,987–75,656,721), was labelled with Cy-3. As a marker for chromosome 5, BAC RP23-153I24 (derived from Chr. 5: 98,182,487–98,393,147), which carries the full-length *Fgf5* gene region (Chr. 5: 98,254,184–98,277,033), was labelled with FITC. These two probes were hybridized to WS chromosomal spreads. Although FITC (green) signals were observed on both copies of chromosome 5, Cy-3 (red) signals were observed on only one copy of chromosome 5. These results suggested that the depigmentation phenotype in WS mice is caused by heterozygous chromosome breakage of 5qc3.3, which includes the *Kit* gene.

**Figure 3:**
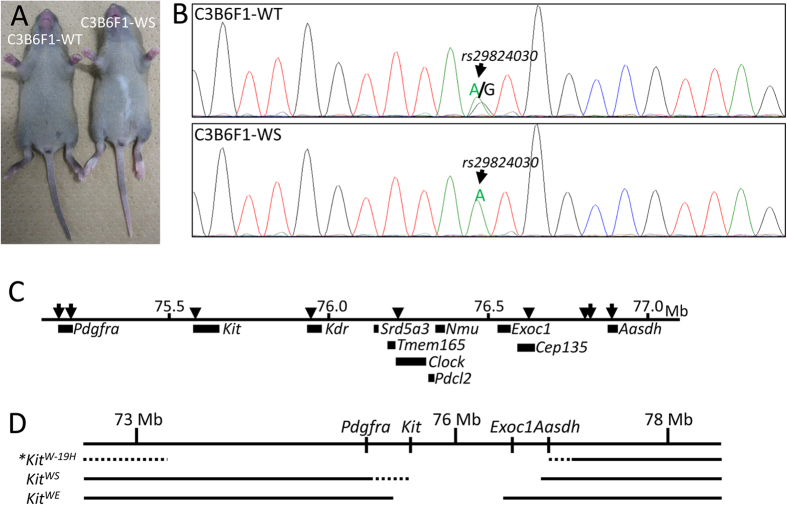
Identification of deletion region in WS. (**A**) C3B6F1-WT and C3B6F1-WS mice were obtained by mating C3H wild-type and WS (C57BL/6 genetic background). The depigmentation phenotype was only seen in C3B6F1-WS. (**B**) Waveform of SNP *rs29824030*. Double peaks (Guanine (G) from C57BL/6J and Adenine (A) from C3H) were detected in C3B6F1-WT. The single A peak was detected in C3B6F1-WS. (**C**) Illustration of the sites of SNPs analyzed in the present study. The arrows indicate the SNP sites detected by double peaks in C3B6F1-WS. The arrowhead indicates the single peak. (**D**) Physical maps of the *Kit*^*W–19H*^, *Kit*^*WS*^, and *Kit*^*WE*^ alleles. The solid line, dashed line, and blank indicate undeleted, unknown, and deleted genomic regions, respectively. *The map of *Kit*^*W–H19*^ is referenced from the report of Nagle^41^.

To identify the deletion region in WS by polymorphism analysis, F1 progeny with normal pigmentation (C3B6F1-WT) and depigmentation (C3B6F1-WS) were produced by crossing WS with C3H/HeJ (C3H) mice (Fig. 3A). In C3B6F1-WS mice, there are no B6J allelic polymorphisms in the deletion region derived from the WS genome. On the other hand, both B6 and C3H allelic polymorphisms were detected in all regions of the genome in C3B6F1-WT. The presence of the B6J genomic region in C3B6F1-WS mice was examined by single nucleated polymorphism (SNPs) analyses. At the *rs29824030* SNP which is located in the *Kit* gene region, we found the C3H genotype only in C3B6F1-WS mice (Fig. 3B). Moreover, we then analyzed SNPs in 5qc3.3. Surprisingly, SNPs from B6J were not detected on *rs33458703*, *rs31548445*, *rs31561146*, or *rs33566798* (Table 1 and Fig. 3C). These results indicated that a genomic region of at least 1.2 Mb containing nine genes (*Kit*, *Kdr*, *Srd5a3*, *Tmeme165*, *Clock*, *Pdcl2*, *Nmu*, *Exoc1*, and *Cep135*) is deleted in WS mice (Fig. 3C & D). This *de novo* large mutant allele was designated as *Kit*^*WS*^.

### Peri-implantation lethality involving *Kit* ^*WS*^

King *et al.*^6^ reported a genomic deletion of more than 3.5 Mb (Chr. 5: 73.2 − 76.9 Mb) in the *Kit*^*W–19H*^ mutant allele (Fig. 3D). Interestingly, homozygous *Kit*^*W–19H*^ mutants (*Kit*^*W–19H/W–19H*^) died around the peri-implantation stage, although *Kit* homozygous null mutant mice (*Kit*^*W/W*^) survived until birth^4^. These reports suggest that the causative gene(s) for peri-implantation lethality is/are located around the *Kit* gene. To understand the characteristics of *Kit*^*WS/WS*^ during embryonic development, *Kit*^*+/WS*^ mice were intercrossed. We first analyzed the *Kit* genotypes in pre-implantation embryos on embryonic day (E) 3.5. Five of 33 blastocysts were *Kit*^*WS/WS*^ and showed no morphological abnormalities. In contrast, no *Kit*^*WS/WS*^ E7.5 embryos (0/24) were found (Table 2). To confirm the embryonic lethality from the 2-cell to blastocyst stages, we collected 2-cell stage embryos from *Kit*^*+/WS*^ intercrosses. Forty-four 2-cell stage embryos were collected from oviducts and cultured in KSOM medium. All embryos were developed to blastocyst. These results indicated that homozygous *Kit*^*WS*^ mutants die at the peri-implantation stage.

**Figure 4:**
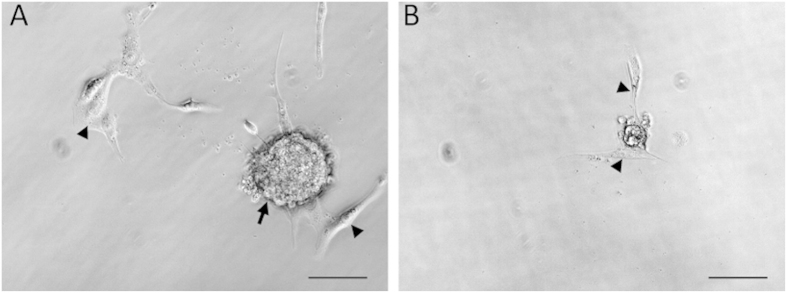
Cultivated *Kit*^*WS/WS*^ blastocysts. (**A**) Dome-shaped ES cell-like colonies (arrow) and TG-like cells (arrowheads) were observed in cultured blastocysts derived from *Kit*^*+/+*^ or *Kit*^*+/WS*^. (**B**) In contrast, although TG cells were seen (arrowheads), no ES cell-like colonies were seen in *Kit*^*WS/WS*^. Scale bar = 100 μm.

Genetic abnormalities in blastocysts are sometimes obscured by maternal factors^8^,^17^. To analyze the *Kit*^*WS/WS*^ phenotype in detail without the effects of maternal factors, we performed *in vitro* cultivation of blastocysts for 1 week. In cultivated blastocysts, embryonic stem (ES) cells and trophoblast giant (TG) cells arise from the inner cell mass (ICM) and trophectoderm (TE), respectively^18^,^19^. Thirty-three blastocysts derived from *Kit*^*+/WS*^ intercrosses were cultured on gelatine-coated dishes. In wild-type or *Kit*^*+/WS*^ blastocysts, both ES cell-like dome-shaped colonies and TG-like cells with an enlarged nucleus were observed (Fig. 4A). Interestingly, no ES cell-like colonies developed from *Kit*^*WS/WS*^ even though TG-like cells were observed (Fig. 4B and Table 3). These results suggest that the early embryonic death in *Kit*^*W/WS*^ is caused by defects in ICM proliferation.

### Association between peri-implantation lethality and *Exoc1* mutation

**Figure 5:**
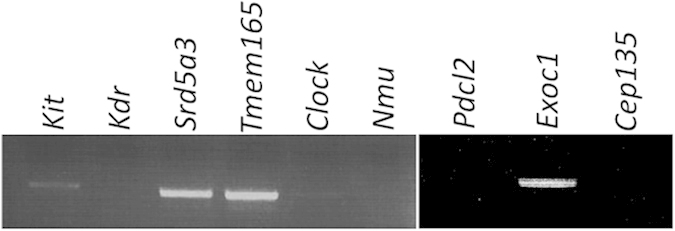
RT-PCR of blastocyst. *Kit*, *Srd5a3*, *Tmeme165*, and *Exoc1* signals were detected on RT-PCR analysis of nine candidate genes using RNAs from wild-type blastocysts.

To narrow down the number of candidate causative genes for peri-implantation lethality, we examined the blastocyst expression of nine genes located in the region of the deletion in *Kit*^*WS*^ (Fig. 3C). On RT-PCR analysis with wild-type blastocysts, signals were detected for *Kit*, *Srd5a3*, *Tmem165*, and *Exoc1*, but not *Kdr*, *Clock*, *Nmu*, *Pdcl2*, or *Cep135* (Fig. 5), suggesting that one or more of the former four genes (*Kit*, *Srd5a3*, *Tmem165*, and *Exoc1*) may be related to peri-implantation lethality.

**Figure 6:**
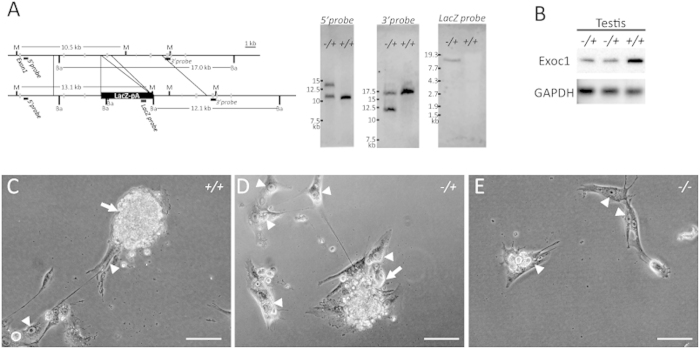
*Exoc1* knockout mouse. (**A**) Southern blotting of *Exoc1*^−*/+*^ and *Exoc1*^*+/+*^ mice. *MfeI*-digested DNA fragments were detected by 5′ outer and *LacZ* inner probes, and the *Bam*HI-digested DNA fragments were detected by 3′ outer probes. (**B**) Western blotting showed that Exoc1 protein expression was reduced in *Exoc1*^−*/+*^ mice. (**C**–**E**) Cultivated blastocysts from *Exoc1*^−*/+*^ intercrosses. TG-like cells with an enlarged nucleus (arrowheads) were seen in cultivated embryos of either genotype. Dome-shaped colonies (arrows) were not seen in *Exoc1*^−*/*−^. M, *Mfe*I; Ba, *Bam*HI; Scale bar = 100 μm.

It has been reported that neither *Kit*, *Srd5a3*, nor *Tmem165* knockout mice died before E7.5^4^,^20^,^21^. We first examined *Exoc1* as the candidate gene. To produce *Exoc1* null mutant mice, an *Exoc1* gene-targeted ES cell line (#78575) was obtained from the European Conditional Mouse Mutagenesis Program (EUCOMM), and heterozygous *Exoc1* mutants (*Exoc1*^−*/+*^) were successfully generated from the ES cells. In *Exoc1*^−*/+*^, the proper targeting allele was confirmed by Southern blotting analysis (Fig. 6A) and Exoc1 protein expression level was shown to be decreased (Fig. 6B).

As expected, no *Exoc1*^−*/*−^ embryos (0/30) were found at the E7.5 stage (Table 4). To investigate whether *Exoc1* plays a critical role in ICM growth of post-blastocyst embryos, we performed *in vitro* cultivation of blastocysts derived from *Exoc1*^−*/+*^ intercrosses. Interestingly, no ES cell-like dome-shaped colonies were observed in any *Exoc1*^−*/*−^ blastocysts (9/9) despite the development of TG-like cells (Fig. 6E and Table 5). In contrast, both ES and TG-like cells were observed in *Exoc1*^−*/+*^ and *Exoc1*^*+/+*^ embryos. To investigate whether Exoc1 is only expressed cells from ICM, we then performed X-gal staining. As expected, the blue color signals were detected in ES cell-like dome-shaped colonies and ES cells but not in TG-like cells (Supplemental Fig. 1). These results indicated that Exoc1 is essential for peri-implantation development, especially for ICM outgrowth in mouse embryos.

### Lack of association between peri-implantation lethality and Mb-scale deletion (from *Kit* to *Nmu*)

The detection of not only *Exoc1* but also *Kit*, *Srd5a3*, and *Tmem165* mRNAs in blastocysts (Fig. 5) suggested that the peri-implantation lethality may also be caused by polygenic effects involving these genes. Therefore, we attempted to produce new gene-modified mice carrying a large deletion mutation extending from *Kit* to *Nmu*, but not *Exoc1*, by CRISPR/Cas9 system.

**Figure 7:**
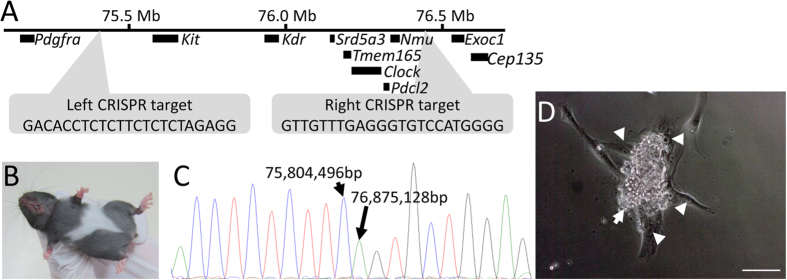
*Kit*^*WE*^ genome edited mice. (**A**) Illustration of CRISPR/Cas9 target sites and genetic regions of candidate genes. The left CRISPR target site was located on Chr. 5: 75,408,459–75,408,481 and the right target site was located on Chr. 5: 76,444,361–76,444,383. The genomic region between left and right targets contained the seven genes, *Kit*, *Kdr*, *Srd5a3*, *Tmeme165*, *Clock*, *Pdcl2*, and *Nmu*. (**B**) The depigmentation phenotype of *Kit*^*WE/+*^ mice was the same as *Kit*^*WS/+*^. (**C**) Genomic sequencing analysis exhibited that the genetic region between 75,804,497 and 76,875,127 on Chr. 5 was deleted. (**D**) Cultivated *Kit*^*WE/WE*^ blastocyst. Both ES (arrow) and TS (arrowheads) like-cells were seen. Sale Bar = 100 μm.

Two CRISPR targets located 240 kb upstream of *Kit* and 81 kb downstream of the *Nmu* gene region were designed separated by a distance of approximately 1.1 Mb (Fig. 7A). Both targeted CRISPR expression vectors were co-microinjected into the pronuclei of 54 fertilized B6J oocytes, and nine neonates were obtained. As expected, one founder mouse exhibited abnormal pigmentation similar to that in *Kit*^*WS/+*^ mice (Fig. 7B). We then confirmed the genomic sequence around the target sites in this mouse. Sequence analysis revealed deletion of a 1.07-Mb genomic region (Chr. 5: 75,804,497–76,875,127) in the founder mouse (Fig. 7C). We named this induced large mutant allele *Kit*^*WE*^ (Fig. 3D). The phenotype of *Kit*^*WE/WE*^ was examined by *in vitro* cultivation of blastocysts derived from *Exoc1*^*WE/+*^ intercrosses. In contrast to *Kit*^*WS/WS*^ and *Exoc1*^−*/*−^, ES cell-like colonies developed from *Kit*^*WE/WE*^ blastocytes (6/8) (Fig. 7D and Table 6). These results indicated that the loss of three genes (*Kit*, *Srd5a3*, and *Tmem165*) expressed in the blastocyst was not associated with the failure of embryonic development at the peri-implantation stage. Therefore, we concluded that *Exoc1* is the causative gene for peri-implantation lethality in *Kit*^*WS/WS*^.

## Discussion

In this study, we found a novel spontaneous mutant mouse with ventral body depigmentation. The results of polymorphism analyses indicated deletion of a genomic region of more than 1.2 Mb containing nine genes (from *Kit* to *Cep135*) in the *Kit*^*WS*^ mutant allele. In blastocyst cultivation experiments, inhibition of ICM outgrowth was seen in both *Kit*^*WS/WS*^ and *Exoc1*^−*/*−^, but not *Kit*^*WE/WE*^. These results suggested that peri-implantation lethality in *Kit*^*WS/WS*^ is caused by monogenic *Exoc1* gene deletion.

In polymorphism analyses with C3B6F1-WS (Table 1 and Fig. 3), only C3H alleles were detected in five SNPs located between *Kit* and *Cep135*. These data clearly indicated that the nine genes are completely deleted in *Kit*^*WS*^, although the deletion ends were not determined. As depigmentation phenotypes in laboratory mice can be found easily, a great variety of genetic mutations in and the around *Kit* gene region have been reported^22^. However, the *Kit*^*WS*^ differs from other *Kit* mutant alleles. Therefore, the *Kit*^*WS*^ is considered to be a *de novo* spontaneous mutation allele.

Of the nine candidate genes (*Kit*, *Kdr*, *Srd5a3*, *Tmeme165*, *Clock*, *Pdcl2*, *Nmu*, *Exoc1*, and *Cep135*), the biological functions of six (*Kit*, *Kdr*, *Srd5a3*, *Tmeme165*, *Clock*, and, *Nmu*) have been analyzed using knockout mice. As described above, *Kit* null mutant mice (*Kit*^*W/W*^) die around the first week of life^4^. *Kdr* knockout mice die between E8.5 and E9.5 due to failure of blood island formation and vasculogenesis^23^. *Srd5a3* knockout mice die between E11.5 and E13.5 with open neural tubes^20^. No embryonic lethal phenotypes were seen in knockout mice for each of the three genes, *Tmem165*^21^, *Clock*^24^, and *Nmu*^25^. Although there have been no previous reports regarding *Cep135* null mutant mice, a 1-bp deletion mutation of *CEP135* was found in humans. This c.970delC mutation generates a non-functional truncated CEP135 protein. Patients homozygous for the c.970delC mutation can survive^26^. Lopez *et al.* reported that *Pdcl2* was only expressed in germline cells^27^. *Pdcl2* mRNA was not detected by RT-PCR in wild-type blastocysts in the present study (Fig. 5). Based on these previous reports and our observations, *Exoc1* emerged as the most likely candidate responsible for the observed peri-implantation embryonic lethal phenotype. In accordance with this hypothesis, the present study exhibited that the abnormal phenotype of *Exoc1*^−*/*−^ embryos was the same as that of *Kit*^*WS/WS*^.

*Exoc1*, which was initially found in the yeast secretory pathway^28^, is a component of the exocyst complex^29^. The exocyst complex plays an important role in the tethering step in exocytosis^30^. As cell membrane proteins and lipids are transported to the cell membrane by exocytosis, the exocyst complex is involved in not only vesicle secretion but also cell adhesion and migration^7^. In fact, Andersen *et al.* reported that the Exoc1-containing exocyst complex was required for assembly of the desmosome, which is one of the key structures in cell–cell adhesion^31^. Furthermore, the exocyst complex is involved in dynamic remodelling of the actin cytoskeleton by regulating Arp2/3-mediated actin polymerization^32^. The peri-implantation lethal phenotypes of mutations in desmosome-related genes (*Dsp*, *Dsc3*, and *Dsg2*) and *Arp3* knockout mice are similar to that of *Exoc1*^−*/*−^, suggesting that *Exoc1* may function in desmosome assembly and/or remodelling of the actin cytoskeleton in early embryo development^8^,^9^,^10^,^11^. In addition, 15% *Kit*^*WS/WS*^ (Table 2) and 11% *Exoc1*^*−/−*^ (Table 5) blastocysts were obtained from *Kit*^*+/WS*^ and *Exoc1*^*−/−*^ intercross, respectively, indicating lower Mendelian ratio than expected. However, all 2-cell embryos from *Kit*^*+/WS*^ intercross showed no developmental failure to the blastocyst stage *in vitro* (data not shown). Therefore, we might also need to investigate the function of Exoc1 in early embryo development prior to the 2-cell stage or in gamete development in future.

We examined the polygenic effects in mice carrying the *Kit*^*WE*^ large deletion induced by the CRISPR/Cas9 system. While the seven genes (from *Kit* to *Nmu*) were completely deleted in *Kit*^*WE/WE*^ blastocysts (Fig. 3D), ICM outgrowth was normal (Fig. 7D). These observations indicated that the seven genes, including *Kit*, *Srd5a3*, and *Tmem165*, expressed in the blastocyst were not involved in monogenic or polygenic defects during peri-implantation embryonic development. In addition, we clearly showed that the CRISPR/Cas9 system was able to induce Mb-scale deletion by zygote microinjection. This technology is expected to produce mice with precise large deletions for investigation of various human diseases associated with chromosome breakage.

In conclusion, we discovered that *Exoc1* null mutation is the cause of peri-implantation lethality. Although the molecular mechanisms of action of Exoc1 are unclear, our findings will contribute to understanding of early embryonic development.

## Materials and Methods

### Animals

Laboratory mice were purchased from Charles River Laboratories Japan. Animal experiments were carried out as described previously^33^,^34^. All animal experiments were carried out humanely in accordance with the Regulations for Animal Experiments of the University of Tsukuba and Fundamental Guidelines for Proper Conduct of Animal Experiment and Related Activities in Academic Research Institutions under the jurisdiction of the Ministry of Education, Culture, Sports, Science, and Technology of Japan and with approval from the Institutional Animal Experiment Committee of our university.

### Western blotting

Western blotting analyses were performed according to standard protocols with antibodies to Kit (3074: CST), Actin (P68133: Millipore), Exoc1 (11690-1-AP: Protein Technologies Ltd.), and GAPDH (FL335: Santa Cruz).

### Genomic PCR & sequencing

Investigation of SNPs and detection of the deletion region in the *Kit*^*WE*^ allele were performed with *PrimeSTAR*^®^ *GXL* DNA Polymerase (Takara) and the primers listed in Supplemental Table 1. An Applied Biosystems® 3500 Genetic Analyzer and a BigDye® Terminator v3.1 Cycle Sequencing Kit (Thermo Fisher Scientific) were used to confirm DNA sequences of PCR products, which were purified with a Fast Gene Gel/PCR Extraction Kit (Nippon Genetics).

### FISH

Generation FITC- and Cy3-labelled BAC DNA probes, preparation of chromosome spreads from *Kit*^*WS/+*^ mice, and probe–chromosome spread hybridization were conducted as described^35^.

### Embryo manipulation & blastocyst cultivation

Superovulation was induced by injection of pregnant mare serum gonadotropin (PMSG) and human chorionic gonadotropin (hCG) with an interval of 48 hours, followed by mating with male mice. The 2-cell embryos were collected by oviduct flushing and cultured in KSOM medium. The zona pellucida of the blastocyst was removed with acid Tyrode’s solution. Then, blastocysts were cultured in medium as described previously^36^ on gelatine-coated dishes.

### RT-PCR

RNA was collected from approximately 100 blastocysts with Sepasol® (Nakalai Tesque) according to the manufacturer’s protocol. cDNA synthesis was performed as described^33^. PCR was performed with AmpliTaq Gold PCR Master Mix (Thermo Fisher Scientific) and various primers (Supplemental Table 2).

### Production of *Exoc1* KO mice

The *Exoc1* gene-targeted mouse ES cells were provided by EUCOMM, International Mouse Phenotyping Consortium (IMPC). The chimeric mice were developed by aggregation of ES cells with morula embryos derived from ICR mice. Southern blotting was conducted as described^35^. X-gal staining of cultivated blastocysts and ES cells were conducted as described^37^.

### CRISPR/Cas9

The *px330* plasmid (Addgene #42230), used as a CRISPR expression vector, was a gift from Dr. Feng Zhang^38^. The left and right 20-nt target sequences (Supplemental Table 3) were inserted into *px330*. The EGxxFP assay^39^ with pCAG-EGxxFP (Addgene #50716) to check cleavage activities of these two vectors was performed (Supplemental Fig. 2) as decribed^34^. These vectors (5 ng/μl each, circled) were co-microinjected into the pronuclei of one-cell-stage embryos derived from C57BL/6J, and the injected embryos were transferred into pseudopregnant ICR mice according to standard protocols^40^.

## Additional Information

**How to cite this article**: Mizuno, S. *et al.* Peri-implantation lethality in mice carrying megabase-scale deletion on 5qc3.3 is caused by *Exoc1* null mutation. *Sci. Rep.* **5**, 13632; doi: 10.1038/srep13632 (2015).

## Supplementary Material

Supplementary Information

## Figures and Tables

**Table 1:** SNP analysis with C3B6F1-WS.

gene	SNP	Locus	C57BL/6J	C3H/HeJ	C3B6F1-WT	C3B6F1-WS
*Pdgfra*	rs13478329	75,192,475	G	A	G/A	G/A
*Pdgfra-Kit*	rs33555487	75,205,382	C	A	C/A	C/A
*Kit*	rs29824030	75,581,667	G	A	G/A	A
*Kdr*	rs33458703	75,943,660	A	G	A/G	G
*Clock*	rs31548445	76,216,518	C	T	C/T	T
*Cep135*	rs31561146	76,627,052	G	A	G/A	A
*Cep135-Aasdh* I	rs33566798	76,802,546	T	C	T/C	C
*Cep135-Aasdh* II	rs33892425	76,820,488	G	C	G/C	G/C
*Aasdh*	rs6257272	76,887,200	C	T	C/T	C/T

**Table 2:** Embryos from *Kit*^*+/WS*^ intercross.

Stage	*Kit*^*WS/WS*^	*Kit*^*+/WS*^*or Kit*^*+/+*^	Total
E3.5	5 (15%)	28 (85%)	33
E7.5	0 (0%)	24 (100%)	24

**Table 3:** Cultured blastocyst derived from *Kit*^*+/WS*^ intercross.

Genotype	ICMoutgrowth	No ICMoutgrowth	Total
*Kit*^*+/WS*^ *or Kit*^*+/+*^	23 (82%)	5 (18%)	28
*Kit*^*WS/WS*^	0 (0%)	5 (100%)	5

**Table 4:** Embryos from *Exoc1*^*−/+*^ intercross.

Stage	*Exoc1*^*−/−*^	*Exoc1*^*−/+*^	*Exoc1*^*+/+*^	Total
E7.5	0 (0%)	24 (80%)	6 (20%)	30

**Table 5:** Cultured blastocyst derived from *Exoc1*^*−/+*^ intercross.

Genotype	ICM outgrowth	No ICMoutgrowth	Total
*Exoc1*^*+/+*^	12 (63%)	7 (37%)	19
*Exoc1*^*−/+*^	10 (48%)	11 (52%)	21
*Exoc1*^*−/−*^	0 (0%)	9 (100%)	9

**Table 6:** Cultured blastocyst derived from *Kit*^*+/WE*^ intercross.

Genotype	ICM outgrowth	No ICMoutgrowth	Total
*Kit*^*+/+*^	11 (92%)	1 (8%)	12
*Kit*^*+/WE*^	12 (55%)	10 (45%)	22
*Kit*^*WE/WE*^	6 (75%)	2 (25%)	8
