# Supplementary material for: Peri-implantation lethality in mice carrying megabase-scale deletion on 5qc3.3 is caused by Exoc1 null mutation
Source: Sci Rep. 2015 Sep 8;5:13632. doi: 10.1038/srep13632 (PMC4562154; doi:10.1038/srep13632)
Supplement: Supplementary Information [file srep13632-s1.doc]

**Supplementary Information**

Peri-implantation lethality in mice carrying megabase-scale deletion on 5qc3.3 is caused by Exoc1 null mutation

Seiya Mizuno^1†^, Takami Kohei^1†^, Daitoku Yoko^1^, Tanimoto Yoko^1^, Tra Thi Houng Dinh^1^, Saori Mizuno-Iijima^1^, Yoshikazu Hasegawa^1^, Satoru Takahashi^1^, Fumihiro Sugiyama^1#^, and Ken-ichi Yagami^1^

^1^Laborarory Animal Resource Center, University of Tsukuba, 1-1-1 Tennodai, Tsukuba 305-8575, Japan

^†^ S.M. and K.T. contributed equally to this study.

^#^Correspondence and requests for materials should be addressed to F.S.


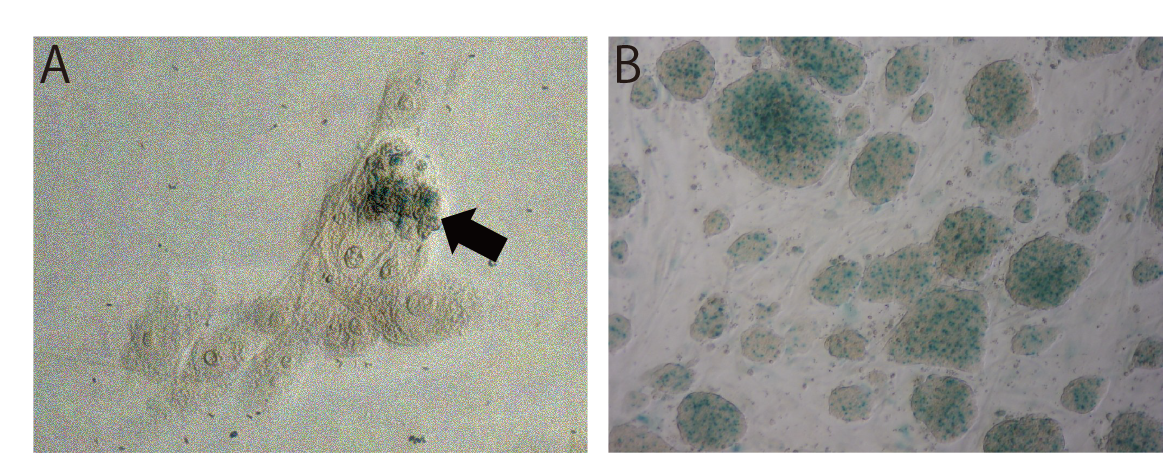


Supplemental Figure 1. X-gal staining of cultivated embryo and ES cells

(A) X-gal staining of cultivated *Exoc1^-/-^* or *Exoc1^-/+^* blastocyst. The blue color singnals were only seen in dome-shaped ES cell-like colony (arrow) but not in TG-like cells. (B) X-gal staining of *Exoc1^-/+^* ES cells (from EUCOMM). The bule color singnals were detected in all ES cell colonies.

Supplemental Figure 2. EGxxFP assay.

px330-left and right, pCAG-EGxxFP-left and right (0.5 mg) plasmids were transfected into HEK293T cells by Lipofectamine® LTX (Life Technologies). After 24 hours, EGFP signals were confirmed. Strong EGFP signals were detected in both px330 and pCAG-EGxxFP co-transfected cells.

| Sup. Table 1. Primers for genomic PCR analyses | | |
| --- | --- | --- |
| rs13478329 | F | AACCACTGAGCCATCTCACC |
|  | R | CTCCTCTGGAACAGGGTCAA |
| rs33555487 | F | TGACCCCCAGCTATAAAACG |
|  | R | TAACCCAAACACAGGCCACT |
| rs29824030 | F | CCAAAGTCCCAAGCTCTGAC |
|  | R | AGGGACATCAAGCCAAACTG |
| rs33458703 | F | CCAAATGCTCCACCAACTCT |
|  | R | GCGGGCTCCTGACTACACTA |
| rs31548445 | F | CACGGTTCCATCATTGTCTG |
|  | R | GACAGCCTGACTGACCCTTC |
| rs31561146 | F | GGGAGGCCTATCTCCAAGTC |
|  | R | CACACAGGCCTTTCCTTCTT |
| rs33566798 | F | CGCAGCTAGGAAGGTTGAGA |
|  | R | TTCTGTGGCCTGATCAGATG |
| rs33892425 | F | CATTGCTGCTGGTCAAGAAA |
|  | R | TTGGCTGCCTACAGGTCTTT |
| rs6257272 | F | ACCTTCAGCCTCCTGAGTGA |
|  | R | TGTCTTTGAAAGGTGGGAAGA |
| KitWE detection | F | CCTACCCTGGTTGCCTCTTATAGCTTCA |
|  | R | TAATCATTTTTCAACCACCCAAAGCAGA |

| Sup. Table 2. Primers for RT-PCR analyses | | |
| --- | --- | --- |
| *Kit* | F | CTGGACCTGGATGATTTGCT |
|  | R | GACTTGGGTTTCTGCTCAGG |
| *Kdr* | F | CCCGCATGAAATTGAGCTAT |
|  | R | GGAATCCATAGGCGAGATCA |
| *Srd5a3* | F | GTCTGCCTTCTTGGTCTTGG |
|  | R | CACCTGGCTTTCTTCACCAT |
| *Tmem165* | F | GGCGACAAGACGTTTTTCAT |
|  | R | AAACGCCAAAAATACGATGC |
| *Clock* | F | GAGGTCGTCCTTCAGCAGTC |
|  | R | TCTGTGGCATACTGGATGGA |
| *Nmu* | F | TGGCATTCTTCCTCCAAAAG |
|  | R | GCCAGAGCTATCGCTGTCAT |
| *Pdcl2* | F | GCTTGGAGGGAAGGAGATGT |
|  | R | CTCAACCATCGCAGAGAATG |
| *Exoc1* | F | CAGGTGCTGAGTGAACTGGA |
|  | R | GGACTGAAGGTGATCCGTGT |
| *Cep135* | F | GTCATTGCCCAGTTGAAGGT |
|  | R | TCATGACGTCTTTGCCTGAG |

| Sup. Table 3. CRISPR target sequences | |
| --- | --- |
| Left side target | GACACCTCTCTTCTCTCTAG |
| Right side target | GTTGTTTGAGGGTGTCCATG |
